# Silica nanoparticle aggregation in calcite replacement reactions

**DOI:** 10.1038/s41598-017-06458-8

**Published:** 2017-11-06

**Authors:** Moritz Liesegang, Ralf Milke, Christine Kranz, Gregor Neusser

**Affiliations:** 1 Institut für Geologische Wissenschaften, Freie Universität Berlin, Malteserstrasse 74-100, 12249 Berlin, Germany; 2 Institute of Analytical and Bioanalytical Chemistry, Ulm University, Albert-Einstein-Allee 11, 89081 Ulm, Germany

## Abstract

Natural nanoparticles are fundamental building blocks of Earth’s bio- and geosphere. Amorphous silica nanoparticles are ubiquitous in nature, but fundamental knowledge of their interaction mechanisms and role in mineral replacement reactions is limited. Here we show how silica nanoparticles replace Cretaceous calcite bivalve shells in a volume- and texture-preserving process. Electron tomography reveals that mineral replacement transfers calcite crystallographic orientations to twinned photonic crystals composed of face-centered cubic silica sphere stacks. During the face-specific replacement process, silica nanoparticles continuously nucleate, aggregate, and form a lattice of uniform spheres parallel to calcite low-energy facets. We explain the replacement process with a new model that unifies recently proposed, probably universal mechanisms of interface-coupled dissolution-precipitation and aggregation-based crystallization; both key mechanisms in geological processes and nanomaterials design and synthesis.

## Introduction

In a first approximation, the Earth’s crust consists of silicate rocks. Chemical weathering reactions of these rocks lead to the *in situ* formation of amorphous silica at silicate mineral surfaces^1^ or release of silica into aqueous fluids, followed by amorphous nanoparticle precipitation through organic and inorganic processes^2–5^. In recent years, amorphous nanoparticles have increasingly been identified as fundamental precursors for crystal formation and growth processes in natural systems^6,7^. These processes are classified as crystallization by particle attachment (CPA) (ref.^8^). However, despite its ubiquitous nature and prototype character for synthetic colloidal crystals^9^, fundamental knowledge of silica nanoparticle formation and ordering processes in nature is limited^4,10^. This knowledge gap also includes the globally widespread interaction of calcium carbonate minerals with amorphous silica^11–13^. Our analytical results and schematic model fill this gap and show that calcite dissolution and silica precipitation are linked by a fluid-mediated replacement process that transfers atomic crystallographic information to an amorphous, mesoscale-ordered material in a natural weathering environment.

## Photonic crystals retain calcite crystallography

**Figure 1:**
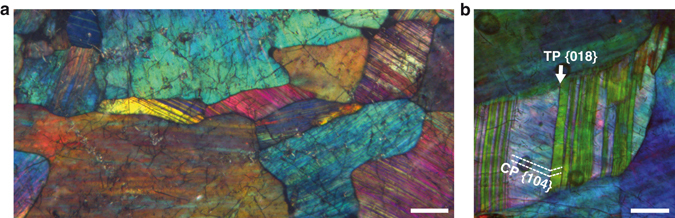
Microphotographs of silica photonic crystals between crossed polarizers. (**a**) Striated photonic crystals with different colors and orientations. (**b**) Crystals display lamellae with periodically alternating colors. Twin (TP) and cleavage (CP) planes are consistent with calcite crystallographic orientations. Scale bars are 100 µm.

**Figure 2:**
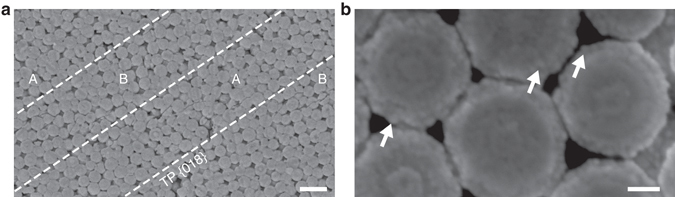
Secondary electron images of amorphous silica sphere geometry and microstructures. (**a**) Polished surface of a twinned crystal with alternating sphere array orientation in an A-B-A-B pattern. Dashed lines highlight the {018} twin planes (TP). (**b**) HF acid-etched spheres show a concentric layering. Arrows highlight ~30 nm-sized subparticles. Scale bars: (**a**) 1 µm, (**b**) 100 nm.

To demonstrate silica nanoparticle interaction and ordering processes, we use silica-replaced bivalve shells collected at Coober Pedy (South Australia) from unsilicified Early Cretaceous Bulldog Shale - a deeply chemically weathered, kaolinitic, marine siltstone (Supplementary Fig. S1). Transmitted light microscopy reveals that the investigated shells consist of a mosaic of anhedral, randomly oriented, optical photonic crystals up to 2.5 mm in size. Microdiffraction data confirm the X-ray amorphous nature of the studied material (Supplementary Fig. S2). The colors of the crystals cover the visible spectrum and upon rotation on the microscope stage, both color and intensity change. Figure 1 shows that observation between crossed polarizers reveals parallel striations (3-32 µm wide) that mimic polysynthetic twin lamellae on trigonal {018} indicative of recrystallized calcite (CaCO_3_). The twinned condition of the lamellae is visible as a periodic color variation in an A-B-A-B pattern. Within some lamellae, thin straight lines intersect with twin planes at an angle of 71.2 ± 1.5° (Fig. 1b). This angle compares closely with the angle between co-zonar calcite {104} cleavage and {018} twin planes at 70.75° (ref.^14^); thus, it evidences that the replacement reaction is intimately coupled to calcite crystallographic orientations. The color variations of the photonic crystals arise from Bragg diffraction effects from colloidal arrays, visualized directly by scanning electron microscopy (SEM), showing that the crystals consist of coherently ordered silica spheres and air-filled pores (Fig. 2a). The sphere arrays perfectly replicate the twin lamellae of calcite in periodically changing orientations. The average sphere diameter varies between samples from 270–400 nm (size dispersion <3.5%). Mild hydrofluoric acid etching of the spheres reveals internal core-shell structures with up to three concentric layers (Fig. 2b). These structures consist of spherical particles 30 ± 2 nm in diameter, which indicates that, at least in the final stage, particle aggregation is the major sphere growth mechanism.

**Figure 3:**
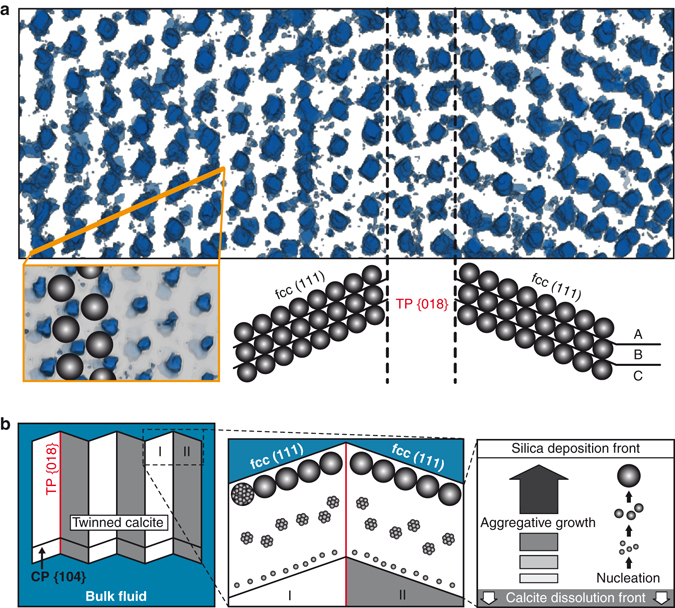
Nanoscale structures and schematic replacement process of twinned calcite crystals. (**a**) Three-dimensional visualization of pores in adjacent twin lamellae (4183 × 1733 nm^2^), projected along uvw [01–1]. The calcite {018} twin plane (TP) separates A B C A B C $\cdots \,$ sphere stacking sequences of fcc lattices. The top view (lower left) shows spheres in the fcc (111) layer (1654 × 1043 nm^2^). (**b**) The orientation of calcite {104} cleavage planes (CP) changes rhythmically between twins I and II. Amorphous silica particles nucleate close to the calcite surface, aggregate in the interfacial fluid film, and self-assemble into close-packed fcc (111) planes.

To evaluate how effectively the templating process captured the crystallographic elements of the calcite precursor, we analyzed the three-dimensional (3D) silica sphere packing and orientation in twin lamellae using focused ion beam (FIB)-SEM nanotomography at a voxel size of 7 × 7 × 20 nm^3^. Figure 3a shows a tomographic image reconstruction of the pore space in adjacent twin lamellae. The uniform pore arrangement indicates uniform sphere arrays. The 3D reconstruction reveals that the lamellae consist of a close-packed lattice of spheres compatible with the face-centered cubic (fcc) structure. Twin and cleavage planes consist of a submicrometer layer of dislocated spheres. These geometric incompatibilities can arise along twin planes by converging sphere stacks and at cleavage planes by stacking faults introduced during growth. To identify the relationship between lattice planes of calcite and silica sphere arrays, we calculated the angles between the calcite {018} twin plane and indexed fcc {hkl} planes by vector analysis. We found that within successive lamellae fcc (111) planes form a zigzag pattern with an angle of 70.1 ± 1.5° to the twin planes. This intersection angle reproduces the rational relation of {018} twin and {104} cleavage planes in calcite and confirms that calcite crystallographic planes template the fcc stacking sequence according to the relationship {104}_calcite_//(111)_fcc_. For preservation in the colloidal crystal, the calcite {104} lattice planes must be thermodynamically most stable and dissolve the slowest. Both theoretical and experimental work^15,16^ support this basic principle, which underlines the importance of anisotropic dissolution rates^17^ to extrapolate laboratory-based results to natural systems.

### The nanoparticle-based replacement process

We combine our results into a generalized model that describes the pseudomorphic replacement of an atomic crystal by the orders of magnitude larger nanoparticle architecture, via a crystallization by particle attachment process (Fig. 3b). To date, abiogenic carbonate-silica replacement has been attributed to infilling of cavities created by bulk dissolution^11^ or silicification controlled by the force of crystallization^12^. Both concepts are inconsistent with the studied material due to (i) the preservation of crystallographic orientation and (ii) the amorphous and nanoporous nature of the replacement silica. In fact, our observations point to a coupled dissolution-precipitation mechanism along a moving reaction interface^18^, controlled by fluid chemistry between the calcite dissolution front, {104}_calcite_, and the silica sphere deposition front, (111)_fcc_.

The highly selective nature of the replacement reaction indicates that calcite dissolution initiates silica precipitation. The dissolution reaction releases Ca^2+^ and CO_3_^2−^ into the fluid film and creates a sharp localized pH and salinity rise^19^ that gradually decreases toward the bulk fluid. This local change lowers the amorphous silica solubility, induces supersaturation and precipitation^20,21^, and confines particle nucleation close to the moving calcite dissolution front. In contrast to the classical LaMer nucleation-and-growth^22^ and Ostwald ripening mechanisms^23^, particle growth in the natural precipitating system is not temporarily limited by the silica monomer concentration and does not compete with nucleation. Thus, particles grow homogeneously along a multistep aggregative pathway of increasingly large units at increasing distance from the calcite surface, in a process analogous to the formation of uniform silica nanocolloids from alkoxysilanes^24^ and hydrothermal brine solutions^4^.

The uniformity of the sphere arrays described herein requires constant physicochemical conditions in the fluid film (e.g. ionic strength, pH, and nucleation/growth rate) throughout the entire replacement process. The nanoporous sphere fcc geometry (~26% porosity) is permissive and enables mass transport and the necessary permanent fluid access to the calcite dissolution front. As soon as the calcite dissolution and silica deposition fronts advance synchronously, a steady state is established and small particles form continuously until the replacement process is completed. Once these particles move due to their Brownian motion, the competition between van der Waals attraction and electrostatic repulsion drives their interaction potential. The ions in the interfacial fluid film can neutralize the particle surface charge, compress the repulsive electrical double-layer, and promote the irreversible aggregation of marginally unstable particles^20^. Electron probe microanalyses show that up to 1.72 wt% of the fluid cations (primarily Al^3+^, Supplementary Table 1), apart from silica, are structurally incorporated during particle growth^10^. The particles in the propagating fluid film continuously collide, stick together, and form larger more stable aggregates. The last active growth step is the aggregation of the observed ~30 nm-sized subparticles that form distinctly larger spheres with a narrow size distribution. The final spheres then self-assemble into minimum energy hexagonal close-packed fcc (111) planes that stack layer-by-layer parallel to the {104} lattice plane of calcite. Ultimately, the colloid undergoes a phase transition into the long-range ordered, thermodynamically favored fcc lattice^9^ that forms the bulk photonic crystal.

### Implications for fluid-mediated replacement reactions

The model proposed herein offers a crystallization by particle attachment process that potentially operates over a wide range of replacement reactions by initially nanocolloidal amorphous and crystalline phases. These replacement reactions include, for example, silica and carbonate pseudomorphism and fossilization^12,25^, or other replacement reactions crucial for palaeoenvironmental isotopic and redox condition studies such as chert nodule^5^ and pyrite (FeS_2_) formation^26^. The oriented attachment of crystalline nanoparticles^8^ can be incorporated into our model and explains the transfer of crystallographic information (epitaxy) across the fluid film in interface-coupled dissolution-precipitation reactions. In addition, our results suggest a potential atomic crystal templating approach for the controllable synthesis of 3D self-assembled functional materials with ordered lattices and a single domain size of several millimeters. In the light of constant progress in high-resolution analytical instrumentation^27^ and ever-increasing evidence of nanoparticles as fundamental building blocks in natural environments^8^, we propose that nanoparticle-based mineral formation is a key mechanism in fluid-mediated replacement reactions in geological processes and the synthesis of advanced functional materials, such as molecular sieves, heterogeneous catalysts, semiconductors, and photonic devices^28,29^.

## Methods

Four bivalve shell samples with their respective host rock were collected in the Allan Rise precious opal field (WGS84 -29.3974°N 134.8581°E) located 52 km south of the Coober Pedy Township (South Australia). The samples were extracted from bulldozer cuts approximately 20 m below the present day surface. Shell samples analyzed are white to milky with moderate play-of-color (POC).

Bivalve shells and their friable porous host rock (Supplementary Fig. S1) were cut using a diamond-impregnated steel lapidary saw cooled with water-free fluid. Diamond polished thin sections (30 µm thick) were prepared using standard procedures. For polarized light microscopy, a Zeiss Axio Lab.A1 petrographic microscope was used. At least 400 intersection angles between straight twin and cleavage planes of crystals were measured and averaged over each sample. Non-destructive µ-XRD was used on thick sections, at the University of Tübingen (Germany), with a modified Bruker AXS micro-X-ray diffractometer D8 discover with focusing X-ray optics and a VANTEC500 2D-detector. Diffractograms (Supplementary Fig. S2) were recorded for 300 s at a beam diameter of 50 µm in the 2θ range 7–67°, using Co-Kα radiation at 30 kV and a tube current of 30 mA. Quantitative element concentrations (Supplementary Table 1) were determined on carbon-coated, polished thin sections, using a JEOL JXA 8200 Superprobe operated at 15 kV acceleration voltage, 20 nA beam current, and a beam diameter of 10 μm. For each specimen, at least 30 crystals with 30 point analyses each were measured. The samples were analyzed for the elements Si, Ti, Al, Fe, Mg, Mn, Ca, Ba, Sr, Na, K, S, and P. Acquisition time for Na analysis was 5 s on peak and 5 s on background. Peak and background of other elements were measured for 10 s each. The instrument was internally calibrated using natural silicate, oxide, and basalt (VG-2) and rhyolite (VG-568) glass.

The morphologies of silica spheres and their subparticles were investigated on surfaces of thin sections and freshly fractured material by SEM. Specimens were etched in 10 vol% HF solution for 15 s, dried, and sputter-coated with ~15 nm W. Secondary electron images were obtained with a Zeiss Supra 40 VP Ultra SEM instrument, at an acceleration voltage 5 kV and beam current of 10 nA. Subparticles were identified with the InLens detection mode. For each specimen, sphere and subparticle diameters have been determined by averaging over at least 1000 manually measured particles. The sphere diameter dispersity has been calculated as relative standard deviation. The sphere stack geometry of adjacent twin lamellae was characterized by FIB-SEM nanotomography of three pre-selected regions on thin sections. The thin sections were carbon-coated (~5 nm) to enhance electrical conductivity and mounted onto a SEM specimen stub. The sample sides were covered with silver glue to improve charge conduction between the top surface and the stub. Focused ion beam (FIB)-SEM nanotomography was conducted with a FEI Helios Nanolab 600 scanning electron microscope equipped with a focused Gallium-ion beam. To protect the sample from beam damage during FIB-milling, the surface was covered twice with a platinum layer using ion beam-induced deposition. A block face was generated by milling a regular cross section perpendicular to the twin plane of adjacent lamellae. After a cleaning cross section the area for the slice and view process was defined. Slice and view was performed using the software Auto Slice & View (FEI). Ion beam milling was conducted at 30 kV with a beam current of ﻿93 pA﻿. Secondary electron images were obtained at an acceleration voltage of ﻿5﻿ kV and beam current of 86 pA. The total number of slices ranged between 140 and 200 per sampled region. With each step, 20 nm of the material was removed and the newly produced surface was imaged with the SEM (Supplementary Video S1) using the secondary electron signal recorded with the through lens detector. The pixel dimensions of a recorded image were set to 7 × 7 nm^2^ (voxel size of 7 × 7 × 20 nm^3^).

Secondary electron images have been processed with the open source software Fiji^30^. We used the SIFT plugin^31^ for automatic alignment of the slice stacks. The aligned stacks were cropped and processed to reduce image noise using a fast Fourier transform filter. Subsequently, a manual grey level thresholding was applied to each image to segment the pore space from the data. Prior to image binarization, this image correction procedure minimized the interfering effects of scan distortions and electron charging during image acquisition. Segmented image stacks were directly volume-rendered by the Voxel function in Avizo 7.0, as shown in Supplemental Figure S3 and Video S2. The hexagonal close-packed lattice planes (Fig. 3) and the twin planes have been determined manually. Due to the possibility of bias in human judgement, we averaged 25 independent manual runs. We calculated the intersection angles between indexed planes from the normal vectors given by the Avizo software and measured them manually in suitable orientations (e.g. uvw [01–1], as shown in Supplemental Figure S3) for comparison. The fcc lattice was confirmed measuring and calculating the angles between indexed planes, for example, (100) ∧ (111) = 55° and (110) ∧ (111) = 35°.

## Electronic supplementary material

Supplementary Information

Supplementary Video S1

Supplementary Video S2
