# Supplementary material for: Silica nanoparticle aggregation in calcite replacement reactions
Source: Sci Rep. 2017 Nov 6;7:14550. doi: 10.1038/s41598-017-06458-8 (PMC5673956; doi:10.1038/s41598-017-06458-8)
Supplement: Supplementary file 1 — Supplementary Information [file 41598_2017_6458_MOESM1_ESM.pdf]

Supplementary Material

Silica nanoparticle aggregation in calcite replacement reactions

Moritz Liesegang*, Ralf Milke, Christine Kranz and Gregor Neussser

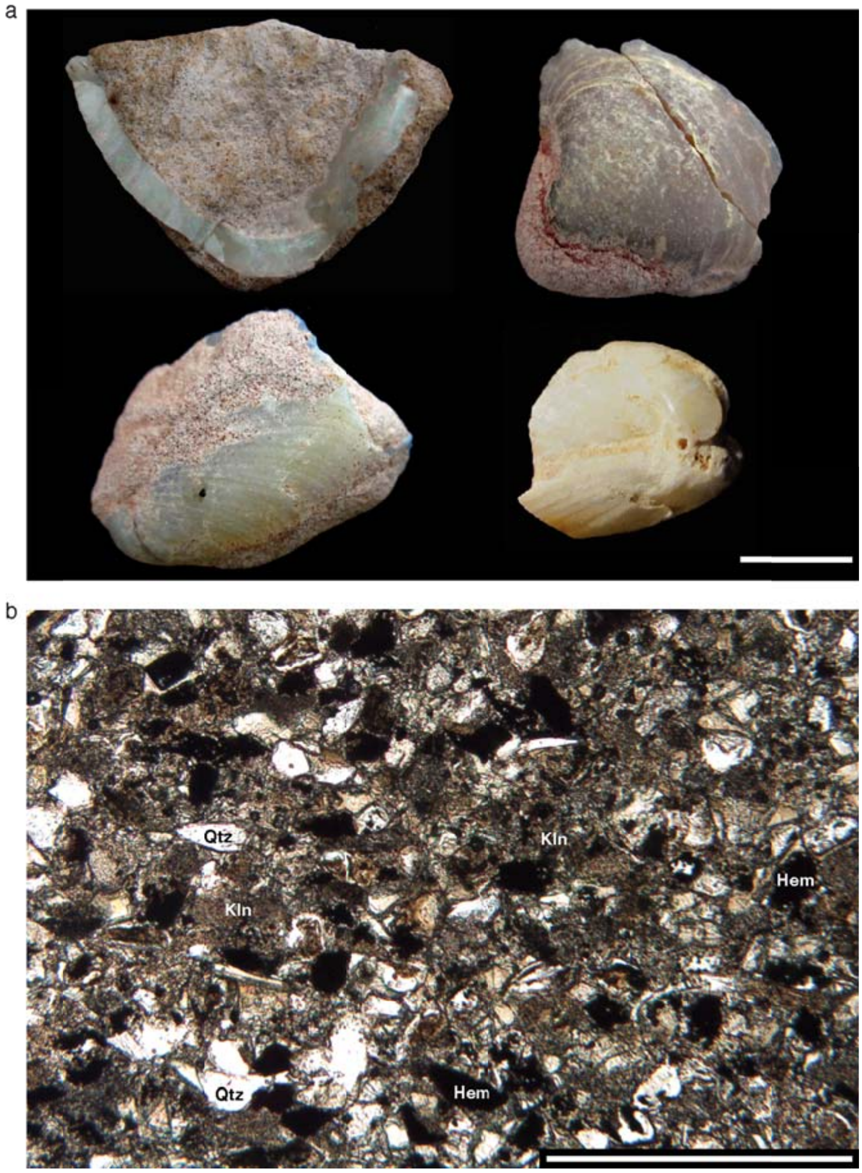

Supplementary Figure S1: Silica-replaced bivalve shells and their host rock. **(a)** Pseudomorphic replacement of the shells preserved delicate details, e.g., growth lines. Unsilicified sediment covers the surface of specimens. **(b)** Microphotograph (plane polarized light) of the deeply chemically weathered Cretaceous Bulldog Shale that hosts the silica-replaced bivalve shells. The unsilicified siltstone consists of kaolinite, quartz, hematite, and minor illite. Scale bars: **(a)** 1 cm, **(b)** 500 µm.

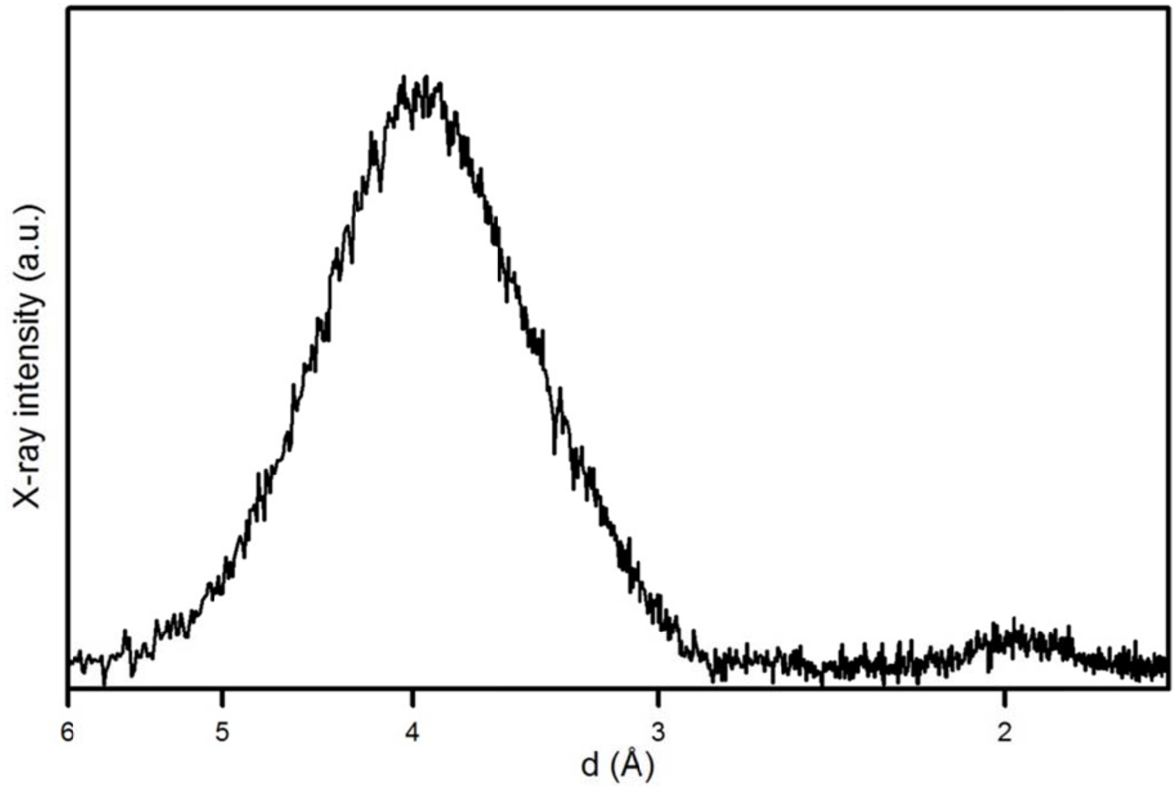

Supplementary Figure S2: A representative μ-X-ray diffractrogram of the replacement silica. All diffractograms show a broad reflection centered at 4 Å, indicative of X-ray amorphous silica[1]. An additional very broad reflection of low intensity occurs at about 2 Å.

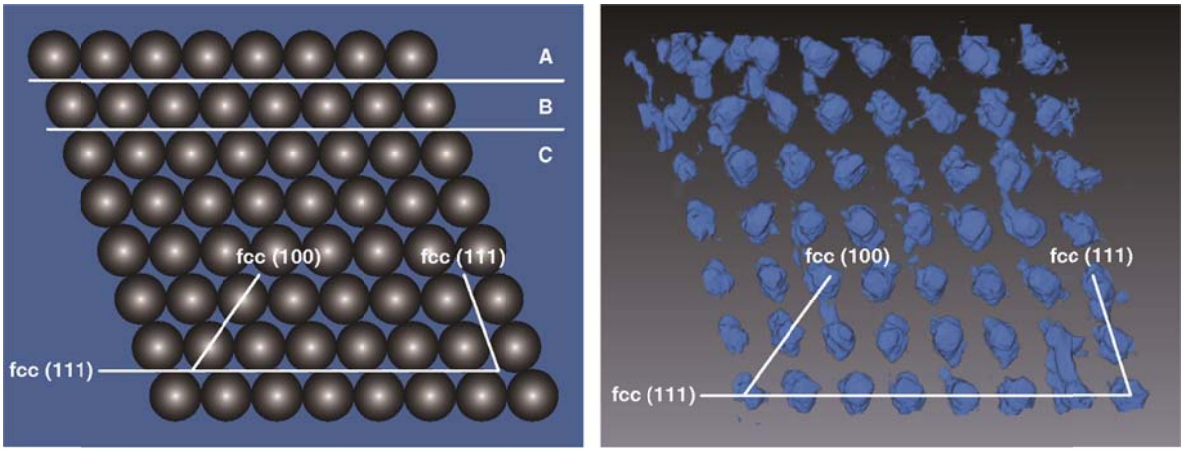

Supplementary Figure S3: Sphere and pore arrangement in an ideal (left side) and the observed (right side) face-centered cubic (fcc) lattice, projected along uvw [01-1]. The fcc lattice consists of an A B C A B C ··· stacking sequence of close-packed (111) planes. Two equivalent (111) and a (100) net plane are shown for comparison in both images.

Supplementary Table 1

Electron microprobe analysis of the investigated shell samples. DL denotes detection limit.

Oxide (wt%)	Sample number			
	1	2	3	4
SiO_2	90.49	91.10	88.91	90.22
TiO_2	<DL	0.05	0.05	<DL
Al_2O_3	1.31	1.50	1.36	1.59
Fe_2O_3-total	0.06	0.08	0.07	0.14
MnO	<DL	<DL	0.06	0.05
MgO	0.03	0.04	0.04	0.04
CaO	0.36	0.41	0.34	0.46
SrO	0.04	<DL	0.03	<DL
BaO	0.02	0.03	0.08	0.08
Na_2O	0.08	0.20	0.07	0.22
K_2O	0.12	0.19	0.11	0.19
SUM total	92.51	93.60	91.12	92.99
SUM impurities	2.02	2.50	2.21	2.77
Impurities (element wt%)	1.22	1.50	1.32	1.72

Captions for Supplementary Video Files S1 and S2

Video S1

Slice series (35 slices) of secondary electron images prepared by the Slice-and-View FIB-SEM process. The dimensions of the images are 7168 x 6188 nm^2.

Video S2

A movie of the 3D tomographic reconstruction of adjacent twin lamellae, rotating 360°. A snapshot from this movie is shown in Fig. 3. The dimensions of the 3D reconstruction are 5670 x 2723 x 2000 nm^3.

Supplementary Reference

1. Jones, J. B. & Segnit, E. R. The nature of opal I. Nomenclature and constituent phases. *J. Geol. Soc. Aust.* **18**, 37-41 (1971).
